# Feeding Entrainment of the Zebrafish Circadian Clock Is Regulated by the Glucocorticoid Receptor

**DOI:** 10.3390/cells8111342

**Published:** 2019-10-29

**Authors:** Elisa Morbiato, Elena Frigato, Alberto Dinarello, Francesca Maradonna, Nicola Facchinello, Francesco Argenton, Oliana Carnevali, Luisa Dalla Valle, Cristiano Bertolucci

**Affiliations:** 1 Department of Life Sciences and Biotechnology, University of Ferrara, 44121 Ferrara, Italy; mrblse@unife.it (E.M.); elena.frigato@unife.it (E.F.); 2 Department of Biology, University of Padova, 35131 Padova, Italy; alberto.dinarello@phd.unipd.it (A.D.); nicola.facchinello@unipd.it (N.F.); francesco.argenton@unipd.it (F.A.); 3 Department of Life and Environmental Sciences, Polytechnic University of Marche, 60131 Ancona, Italy; f.maradonna@univpm.it (F.M.); o.carnevali@univpm.it (O.C.)

**Keywords:** glucocorticoid receptors, zebrafish, circadian clock, clock gene, entrainment, feeding, metabolism, *Danio rerio*

## Abstract

Glucocorticoids (GCs) are steroid hormones mainly acting as key regulators of body homeostasis and stress responses. Their activities are primarily based on the binding to the GC receptor (GR), a member of the nuclear receptor family, that regulates tissue-specific sets of genes. GCs secretion follows a circadian rhythmicity with a peak linked to the animal’s activity phase. In mammals, GCs are also implicated in feeding entrainment mechanisms as internal zeitgeber. Here, we investigated, by means of behavioural and molecular approaches, the circadian clock and its regulation by light and food in wild-type (WT) and null glucocorticoid receptor (*gr*^−/−^) zebrafish larvae, juveniles and adults. In both WT and *gr*^−/−^ larvae and adults, behavioural activity and clock gene expression were entrained to the light–dark (LD) cycle and rhythmic in constant conditions. Differences in the pattern of clock genes’ expression indicated a modulatory role of GCs. A significant role of Gr was detected in the feeding entrainment which was absent or markedly dampened in mutants. Furthermore, the expression of two clock-regulated genes involved in glucidic and lipidic metabolism was altered, highlighting the participation of GCs in metabolic processes also in fish. Taken together, our results confirmed the role of GC-mediated Gr signaling in the feeding entrainment in a non-mammalian species, the zebrafish.

## 1. Introduction

Most of the organisms living on the Earth have adapted to daily environmental changes [1]. As a consequence, many physiological and behavioural processes are temporally organized and follow the 24 h cycle. The mechanism that allows organisms to track the time of day and, therefore, to anticipate daily changes is termed circadian clock [1].

Circadian clocks are endogenous intracellular mechanisms based on overlapping molecular feedback loops that generate a self-sustained molecular oscillation of about 24 h, continuously synchronized (*entrainment*) by environmental signals [2]. Among environmental signals (so-called *zeitgebers*, or time givers) the daily light-dark and temperature cycle, and the food availability are the strongest (and well-investigated) zeitgebers able to reset the phase of the circadian clock [2,3,4,5].

In vertebrates, circadian clocks are organized in a multi-oscillatory network comprising a central oscillator, located in the brain (i.e., the suprachiasmatic nuclei (SCN) of the hypothalamus in mammals and reptiles, the pineal gland in birds and reptiles), and many peripheral oscillators in other brain nuclei and organs, such as lateral eyes, liver, heart, lung and kidney. Peripheral oscillators, and the derived cell lines, are capable of persistent rhythmicity, independent of inputs from the central oscillators [6,7]. Molecular mechanisms of the circadian clock are based on positive and negative transcriptional/translational feedback loops [8]. The positive loop involves the transcription factors BMAL1 (also known as ARNTL1a) and CLOCK (or NPAS2) that form heterodimers and act as transcriptional activators of *per* and *cry* genes. The PER:CRY heterocomplexes translocate into the nucleus and inhibit the transcriptional activity of the CLOCK(NPAS2):BMAL complex and thus their own transcription. A second feedback loop involves the orphan nuclear receptors, REV-ERBα/β (also known as NR1D1/2) and RORα that compete to inhibit or promote the *bmal* rhythmic expression [9]. Oscillations in gene expression resulting from these feedback loops have period lengths of approximately 24 h, thus giving rise to overt circadian rhythms. Sequences and functions of most clock genes have been maintained across a broad range of species from invertebrates to humans showing that the basic properties of the circadian clock have been conserved during evolution [8,10].

Many endocrine glands release hormones in the circulatory system with marked daily level variations [11] that play a role in the temporal coordination of biological processes across the body. Among them, glucocorticoids (GCs) and melatonin rhythms have been widely investigated. GCs, such as cortisol and corticosterone, are steroid hormones involved in several physiological processes including metabolism homeostasis, brain functions and immune and stress response [12]. The concentration of circulating GCs is normally regulated by the hypothalamus–pituitary–adrenal (HPA or hypothalamus–pituitary–interrenal, HPI in fish) neuroendocrine axis in response to different kind of stimuli. Besides variations due to stress, plasma GC levels show a daily pattern with a peak prior or in correspondence to the onset of the active phase (i.e., early morning in human and zebrafish and early evening in rodent) [13,14]. In mammals GC rhythmicity is driven by the SCN and entrained to the light-dark cycle perceived by melanopsin-containing retina ganglion cells [15]. GCs have previously been implicated in the feeding entrainment mechanisms and as zeitgeber of peripheral clocks in mammals and in the regulation of circadian cell cycle rhythms in zebrafish [16,17].

Different investigations in mammals indicate that food- and light-entrainable oscillators (FEO and LEO, respectively) are located in different brain structures. Although the SCN are doubtless the mammalian master clocks, other brain nuclei, such as paraventricular and arcuate nuclei of the hypothalamus, play a crucial role in the regulation of feeding entrainment and are regulated by metabolic hormones, such as GCs and insulin [12]. 

The circadian control of feeding has been demonstrated also in fish [18]. Feeding experiment in teleosts [19,20,21,22] showed that fish prefer to feed in one of the phases of the light–dark (LD) cycle. Circadian nature of this feeding rhythm is confirmed by its persistence under constant conditions [23]. The entrainment of behavioural rhythms to periodic food availability in fish, as in mammals, is demonstrated by food anticipatory activity (FAA): the evident increase of locomotor activity in anticipation of the forthcoming meal [3,18]. Data on the neuroanatomical location of FEO in fish are scarce and the molecular mechanisms remain unclear. However, the entrainment of the molecular clock to feeding has been reported in several fish species. Recent investigation in the goldfish *Carassius auratus* showed an effect of the dexamethasone, a glucocorticoid analog, on the expression of several clock genes in the liver [24]. Although limited in number, these studies seem to indicate a conserved role of GCs as regulatory signals of peripheral oscillators in both mammals and fish.

To better understand the role of GCs in the circadian timekeeping system, we deeply investigated behavioural and molecular circadian rhythms in zebrafish. Zebrafish is a valuable model for this topic because: (i) its molecular circadian clock and behavioural circadian rhythms have been well characterized; (ii) it has both FEO and LEO; (iii) its peripheral circadian clocks are directly entrained by light; (iv) the promoter regions of many clock genes have GC-responsive elements (GREs) [7,18,23].

For this aim we studied the circadian clock and its regulation by light and food in a previously established zebrafish null glucocorticoid receptor *gr^ia30/ia30^* (hereafter referred to as *gr*^−/−^ line) mutant line [25]. By contrast with *GR*-null mice [26], zebrafish *gr*^−/−^ mutants survive till adulthood, although with reduced fitness respect to wild-type (WT) fish, and thus it can provide a useful model to study in vivo GC functions. The zebrafish line *gr*^−/−^ displays physiological responses linked to GC-resistance, such as overstimulation of basal HPI due to failure of the negative feedback loop mediated by the GC-Gr complex, resulting in increased cortisol concentration. Moreover, HPI of mutants is also unresponsive to a mechanical stressor [25].

In the present study we compared light and food behavioural entrainment and the expression of clock genes in larvae and adults of *gr*^−/−^ and WT (*gr*^+/+^). A strong photic entrainment at the molecular and behavioural level was evident in larvae and adults from both genotypes. By contrast, we revealed an impairment of food entrainment of the locomotor activity in *gr*^−/−^ larvae and adults. Interestingly, a molecular counterpart of this behavioural phenomenon, such as change in clock gene expression, was not identified.

## 2. Material and Methods

### 2.1. Ethics Statement

All husbandry and experimental procedures were performed in accordance with European Legislation for the Protection of Animals used for Scientific Purposes (Directive 2010/63/EU) and the Italian (D.lgs. 26/2014) animal protection standards. Research was approved by the University of Padova and University of Ferrara Institutional Animal Care and Use Committee and the Italian Ministry of Health (auth. num. 112/2015 and 801/2017-PR).

### 2.2. Zebrafish Rearing

Wild-type (WT) and mutant zebrafish as well as zebrafish of the (9×GCRE-HSV.Ul23:EGFP)ia20 transgenic line (hereafter referred to as ia20 line) [27] were staged and maintained according to standard procedures [28]. Embryos were obtained by natural mating and raised at 28 °C in Petri dishes containing fish water (FW, 50×: 25 g Instant Ocean, 39.25 g CaSO_4_ and 5 g NaHCO_3_ for 1 L) and kept in a 12:12 LD cycle. Larvae and adults were euthanized with an overdose of tricaine methane sulfonate (Sigma-Aldrich, Milan, Italy).

### 2.3. Recording of Adult Locomotor Activity

Adult *gr*^−/−^ and *gr*^+/+^ zebrafish locomotor activity was recorded continuously by means of an infrared photocell (E3S-AD62, Omron, Kyoto, Japan) placed at the aquarium wall. The photocell was placed 2 cm from the water surface and 8 cm from the bottom. The number of light-beam interruptions was counted and stored every 10 min by a computer connected to the photocell. Zebrafish (n = 6–8 per aquarium; 3 aquaria per genotype) were exposed under 12:12 light-dark (12:12 LD) cycle or constant dim light (LL). It is conventional to divide the 24-h LD cycle into 24-h zeitgeber time (ZT) units and indicate the time of lights on as ZT0 and the time of lights off as ZT12. The 24 h circadian cycle is divided into 24-h circadian time (CT) units. As a reference point, in LL, the phase that would normally (i.e., in LD 12:12) coincide with the onset of light, which is called CT0, is conventionally used. In all tests with adults light-emitting diodes (Superlight Technology Co. Ltd., Wuxi, China) sources were used. Irradiance was measured with a radiometer (DO9721, Probe LP9021 RAD, Spectral range 400–950 nm, DeltaOHM, Padova, Italy) and set at 0.6 W/m^2^ for the LD tests and at 0.05 W/m^2^ for the LL tests. The temperature was held constant at 28 °C by means of water heaters (50 W, Sera GmbH, Immenhausen, Germany) and recorded every 10 minutes with data loggers (Hobo Pendant, Onset Computer Corporation, Bourne, MA, USA). The representation of actograms was performed using the chronobiology software El Temps (version 1.228). For each genotype, tests were conducted three times.

### 2.4. Recording of Larvae Locomotor Activity

*gr*^−/−^ and *gr*^+/+^ embryos were collected immediately after spawning and raised in FW in a light- and temperature-controlled incubator. At 4 days post-fertilization (dpf) larvae were placed in 48-well plate (1 larva per well, 1.6 mL of FW medium) in the observation chamber of the DanioVision tracking system (Noldus Information Technology, Wageningen, The Netherlands). DanioVision is equipped with an infrared (IR)-sensitive camera, a temperature controller unit and a power supply to control light intensity. From 5 dpf larvae locomotor activity was tracked for 4 consecutive days and then analyzed by Ethovision 11 software (Noldus Information Technology, Wageningen, The Netherlands). The IR-sensitive camera was set to 25 frames per second. Locomotor activity of each larva was calculated as the total distance moved during a 6-min time window. A minimal distance moved of 0.2 mm was used. Larvae were kept under 12:12 LD cycles (lights on at 06:00, lights of 18:00) or under LL conditions. For light sources, an array of LED strips was used, and irradiances were set at 0.17 W/m^2^ for the 12:12 LD cycle and at 0.015 W/m^2^ for the LL conditions. For each genotype, three biological repeats were conducted.

### 2.5. Feeding Entrainment

To study the effect of feeding time as an exogenous zeitgeber [20] adult *gr*^−/−^ and *gr*^+/+^ zebrafish (N = 8/aquarium; 3 aquaria for each genotype) were maintained under constant darkness (DD) and fed once a day (0.3% of body weight) at a fixed time (i.e., 06:00, 12:00 or 24:00) with dry food (TetraMin, Tetra GmbH, Offelten, Germany) using a programmable feeder (Eihem, Deizisau, Germany). Fasting and shifting of the feeding time were applied to confirm the entrainment. Locomotor activity was recorded continuously by means of an infrared photocell (E3S-AD62, Omron) for 30 days. Two biological repeats were conducted.

The feeding entrainment were also tested in early juvenile *gr*^−/−^ and *gr*^+/+^ zebrafish. Fifteen dpf old larvae from both genotypes were maintained in 10 liters aquaria under LL (0.015 W/m^2^) and constant temperature (28 °C). Larvae/juveniles were daily fed at midday with frozen *Artemia* naupli and powered food (GEMMA Micro 75, Skretting Italy, Mozzacane, Italy). They had free access to the food for one hour. After 20 days of feeding entrainment, early juveniles were placed in a 9-well plate (1 larva per well, 5 mL of FW medium) in the DanioVision tracking system (Noldus Information Technology, Wageningen, The Netherlands) in the same lighting and temperature conditions. Locomotor activity was videotracked for 84 consecutive hours and then analyzed by Ethovision 11 software (Noldus Information Technology, Wageningen, The Netherlands). The IR-sensitive camera was set to 25 frames per second. Locomotor activity of each juvenile was calculated as the total distance moved during a 6-min time window. A minimal distance moved of 0.2 mm was used. For each genotype, five biological repeats were conducted.

### 2.6. Gene Expression Analysis

*gr*^−/−^ and *gr*^+/+^ larvae were maintained under 12:12 LD cycles and sampled at different time points (ZT3, 9, 15 and 21; ZT0 = lights on, ZT12 = lights off), during day 5, 6 and 12 dpf. For each ZT, 15 larvae were sampled and pooled (n = 4 pooled samples per ZT). Juveniles from both genotypes were sampled at CT3, 9, 15 and 21 (For each ZT, 15 larvae were sampled and pooled (n = 4 pooled samples per CT). *gr*^−/−^ and *gr*^+/+^ adult zebrafish livers and eyes were harvested from ZT3 every 6 h for a day (n = 5 per ZT). Total RNA was isolated from zebrafish larvae and tissues using Trizol reagent (Invitrogen, Carlsbad, CA, USA) following the manufacturer’s instructions. The amount, quality and composition of isolated RNA were analyzed by BioSpec-nano (Shimadzu, Kyoto, Japan). One microgram of total RNA was incubated with DNase I (Invitrogen, Carlsbad, CA, USA) at room temperature for 30 min and then at 85°C for 15 min to inactivate the enzyme. DNase-treated RNA was used to perform cDNA synthesis in a final volume of 20 μl, using iScript cDNA Synthesis Kit (Bio-Rad Laboratories, Hercules, CA, USA). The reaction was performed at 42 °C for 30 min, followed by an inactivation step of 5 min at 85 °C. Three microliters of 1:10 diluted first-strand cDNA was PCR amplified with a Chromo4 Real-Time PCR Detection System (Bio-Rad Laboratories, Hercules, CA, USA) using SsoFast EvaGreen Supermix (Bio-Rad Laboratories, Hercules, CA, USA). Thermal cycling conditions were as follows: 3 min denaturation at 95 °C, followed by 40 cycles of a 15 s denaturation step at 95 °C and an annealing-elongation step for 20 s at 60 °C. After amplification, a melting curve analysis to confirm the specificity of the amplicon was performed. Gene-specific primers for *per1b*, *per2*, *clock1a*, *cry1a*, *arntl1a*, *nr1d1*, *pck2* and *srebp1* are reported in Table S1. We verified the efficiency of the primers by constructing standard curves for all genes investigated. The relative levels of each sample were calculated by the 2^−ΔΔCT^ method where CT is the cycle number at which the signal reaches the threshold of detection [29]. As housekeeping genes we used *gapdh, rplp0, 18S* and *rpl13a*, validated reference genes for zebrafish [30,31]. Primers for housekeeping genes are reported in Table S1. Nearly identical results were observed with all housekeeping genes. Each CT value used for these calculations is the mean of three replicates of the same reaction.

### 2.7. Whole-Mount In Situ Hybridization (WMISH)

Zebrafish larvae of the ia20 line were fixed overnight in 4% paraformaldehyde (PFA, Sigma) in phosphate-buffered saline (PBS) at the required stages of development. WMISH for EGFP expression were performed with the probe and condition previously described [27].

### 2.8. Birefringence Assay

Muscle fiber integrity or overall muscle dimension could be measured by birefringence, a light effect determined by the diffraction of polarized light through the pseudo-crystalline arrangement of the muscle myofibril. In this assay muscle fibers appear bright in a surrounding dark environment. Muscle birefringence was analyzed by placing anesthetized larvae mounted in 2% methylcellulose on a glass polarizing filter and covering them with a second polarizing filter on a Leica M165 FC microscope. Embryos were photographed with a Nikon DS-Fi2 digital camera in bright field. The top filter was twisted until it was possible to see the light refracting through the striated muscle. Pixel intensity in the trunk region was measured with ImageJ software. Values were normalized for whole body area of each larvae. Three independent clutches of 10 larvae per genotype were analyzed at 3, 4, 5, 6 and 10 dpf.

### 2.9. Statistical Analysis

All the results were expressed as means ± SEM. Data were analyzed by parametric and non-parametric tests to determine significant differences using the software Prism 5 (GraphPad Inc., La Jolla, CA, USA) or R 3.6.1 (https://www.R-project.org/). *p* values <0.05 were considered statistically significant. Daily and circadian expression gene expression profile (periodicity and phase of oscillation) was evaluated by the RAIN (Rhythmicity Analysis Incorporating Nonparametric) algorithm [32]. The presence of daily and circadian periodicity in the activity rhythms was determined by means of Cosinor [33] or χ^2^ periodogram analysis (ActogramJ 1.0). Periodogram analyses were performed with intervals of 6–10 days. The daily acrophase (i.e., the time at which the peak of a rhythm occurs) of the locomotor activity rhythm was calculated (ActogramJ 1.0) and the average acrophase was determined by vector addition. The Rayleigh test was used to test whether the acrophases deviated from uniform and whether they were concentrated at a given time of the day (*p* < 0.05). Hotelling’s paired test was performed to test for differences among average acrophases of different periods (*p* < 0.05) [34].

## 3. Results

**Figure 1:**
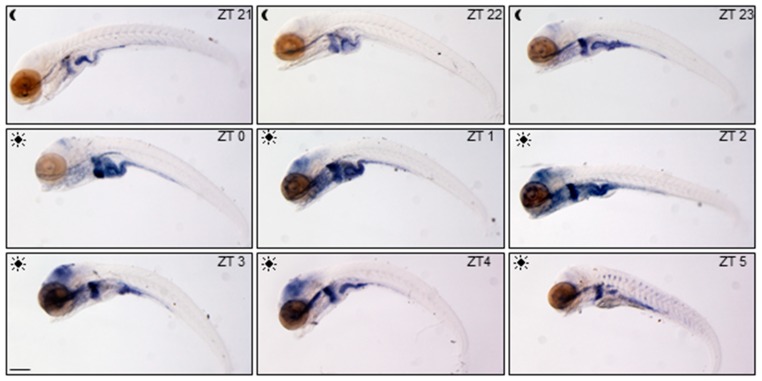
Spatial and temporal variations of glucocorticoid activity. WMISH of *egfp* mRNA in 5-dpf ia20 larvae exposed to standard photoperiodic regime and analyzed from 3 h before to 5 h after light onset (from ZT21 to ZT5). All larvae are lateral views with head pointing to the left. Scale bar: 200 µM.

Spatial and temporal variations of glucocorticoid activity have been firstly detected in the whole larvae using the ia20 zebrafish transgenic line [27]. In this line, enhanced-GFP (EGFP) expression is driven by nine GRE tandem repeats allowing to dynamically trace GC transcriptional activity during development and adult life. We focalized our analysis on *egfp* mRNA WMISH in ia20 larvae during the day starting from ZT21, three hours before the lights on, until ZT5 (Figure 1), in order to visualize the highly dynamic daytime GC activity. GC activity was low and mainly limited to the digestive tract at the end of the scotophase (from 5 to 7 am), but was markedly intensified in the liver and the intestine just before the lights came on, where it remained high till 10 am. In the eyes and the brain the signal increased after lights on from ZT1 to ZT4 and then decreased (Figure 1).

**Figure 2:**
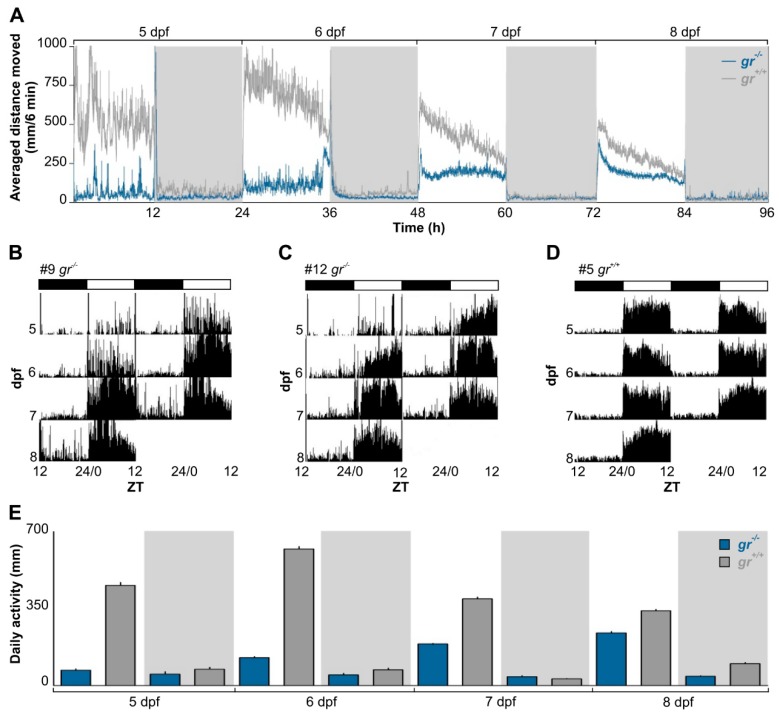
Daily activity rhythms of *gr*^+/+^ and *gr*^−/−^ larvae zebrafish. (**A**) Mean waveform of locomotor activity under 12:12 LD cycles from 5 to 8 dpf (n=120/genotype). Vertical axis shows averaged distance moved (mm/6 min) while x axis indicates time in recording. White and grey bars show light and dark phase, respectively. Data are expressed as mean ± standard error of the mean (SEM). (**B**–**D**) Representative actograms of 3 zebrafish (*gr*^−/−^: **B**–**C**; *gr*^+/+^: **D**). Actograms are double plotted on a 48 h time scale to help the interpretation. The height of each point represents the distance travelled in 6 min. Bars at the top of each actogram indicate light (white) and dark (black) phases the LD cycles. The age of zebrafish is indicated on the left and the zeitgeber time (ZT) is plotted on the bottom of each actogram. (**E**) Mean activity (n = 120/genotype) in the light and dark phases from 5 to 8 dpf. Data are expressed as mean ± SEM.

To determine the role of Gr signalling in generating and regulating circadian rhythms, we firstly analyzed the rhythmic locomotor activity of *gr*^−/−^ and control larvae under various photic conditions using an automated high-throughput videotracking system. Control larvae displayed a daily rhythm of locomotor activity from 5 dpf (Cosinor, *p* < 0.001) and the typical diurnal pattern of zebrafish, with higher activity during the light phase (Figure 2A,D). By contrast, daily rhythms of activity in *gr*^−/−^ larvae became significant one day later, at 6 dpf (Figure 2A–C; Cosinor, *p* < 0.001). Indeed, the difference in the quantity of activity between light and dark phase in mutant appeared from 6 dpf (Tukey’s multiple comparison test, *p* < 0.05), while in control larvae it was already presents at 5 dpf (Tukey’s multiple comparison test, *p* < 0.001; Figure 2E). Furthermore, larvae showed differences in the overall daily amount of activity (Figure 2E; Kruskall–Wallis one-way analysis of variance (ANOVA), *gr*^−/−^: F_(7, 959)_ = 640.8, *p* < 0.0001, *gr*^+/+^: F_(7, 959)_=804.9, *p* < 0.0001) for the whole recording.

**Figure 3:**
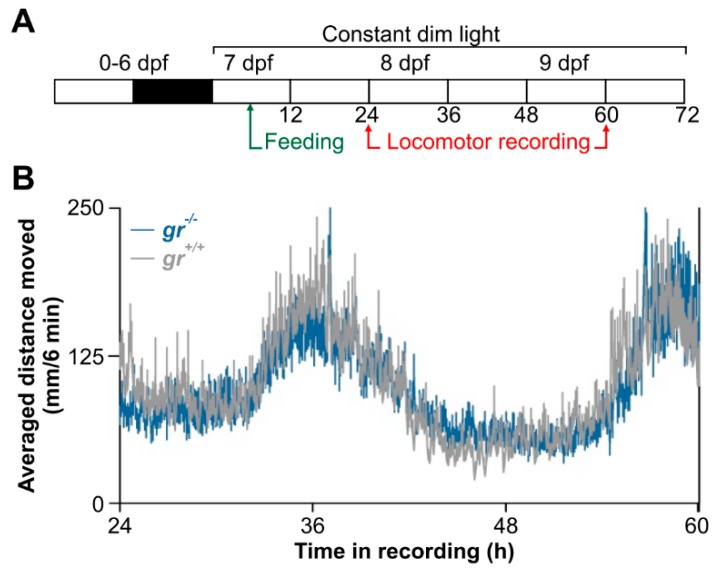
Circadian activity rhythms of *gr*^+/+^ and *gr*^−/−^ larvae zebrafish. (**A**) Larvae were reared under light–dark (LD) cycle from 0 to 6 dpf and then maintained for 3 days in constant dim light (LL). Locomotor activity were recording from 24 to 60 h in LL. (**B**) Mean waveform of locomotor activity under LL for 36 h (n = 144/genotype).

To confirm the presence of a circadian timekeeping system in zebrafish lacking Gr we recorded circadian locomotor activity in larvae kept in constant dim light (LL). Larvae were exposed to 12:12 LD cycle until 6 dpf and then to LL for 3 days (Figure 3A). We recorded the locomotor activity the second and the third day in constant condition and found a clear circadian rhythmicity with a period of about 24 h in both genotypes (Figure 3B; Cosinor, *p* < 0.05).

**Figure 4:**
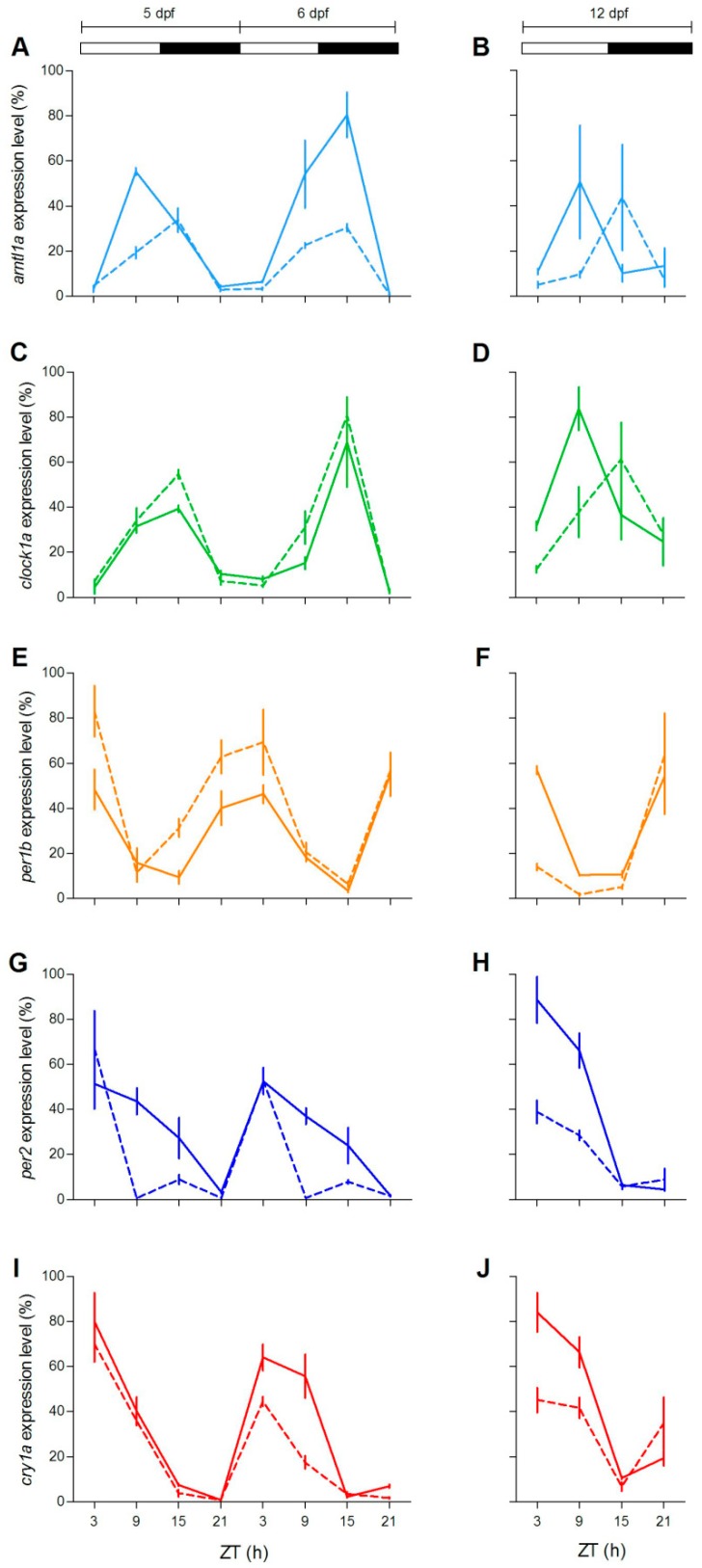
Daily expression levels of clock genes in zebrafish larvae. Quantitative polymerase chain reaction (qPCR) analysis of clock and light-regulated clock gene expression at 5–6 dpf (**A**,**C**,**E**,**G**,**I**) and 12 dpf (**B**,**D**,**F**,**H**,**J**) in zebrafish larvae exposed to LD cycles. For all panels, each point represents the mean ± SEM (n = 4). On the y-axes are plotted relative expression levels (100% is the maximum level detected for each gene in all dpf), while on the x-axes time is expressed as zeitgeber time (ZT, where ZT0 represents lights on). White and black bars represent light and dark periods, respectively. *gr*^+/+^ = solid line; *gr*^−/−^ = dotted line.

To check at the molecular level the lack of behavioural rhythmicity in mutant zebrafish at 5 dpf, we examined expression pattern of a set of clock and clock-related genes at different stages (5, 6 and 12 dpf) under LD cycles. We firstly measured the expression of clock-regulated (*clock1a*, *arntl1a*, *per1b*) and light-regulated (*per2*, *cry1a*) genes [20,35,36]. Larvae from both genotypes showed daily changes of expression levels in all genes investigated (Figure 4; Table S2). However, differences in the expression levels (Figure 4A,E,G,H,J) or in the phase of peaks (Figure 4B,D) have been found for both positive (*arntl1a* and *clock1a*) and negative (*per1*, *per2a* and *cry1a*) elements of the molecular clock. Interestingly, at 12 dpf the increase of expression level at ZT3 is significantly dampened in two light-inducible genes, *per2* and *cry1a* (Figure 4H,J; Pairwise comparison after a Kruskal–Wallis test, *p* < 0.05), and in *per1b* (Figure 4F; pairwise comparison after Kruskal–Wallis test, *p* < 0.05).

To test if the delay of the onset of the daily locomotor rhythmicity of *gr*^−/−^ larvae is linked to physical characteristics of the mutant larvae, we analyzed their body length and muscle birefringence (Figure S1). Together with very faint (*p* < 0.05 only at 3 and 4 dpf; through Mann–Whitney U-test) differences of larvae length, we found a reduction of striated muscle formation in mutants between 3 and 5 dpf with respect to the WT (*p* < 0.05; Mann–Whitney U-test). Birefringence levels were instead comparable between WT and mutant larvae at 6 and 10 dpf (*p* > 0.1; Mann–Whitney U-test). The reduced birefringence was not associated to the patch-like pattern that is normally linked to disorganization of skeletal muscle.

**Figure 5:**
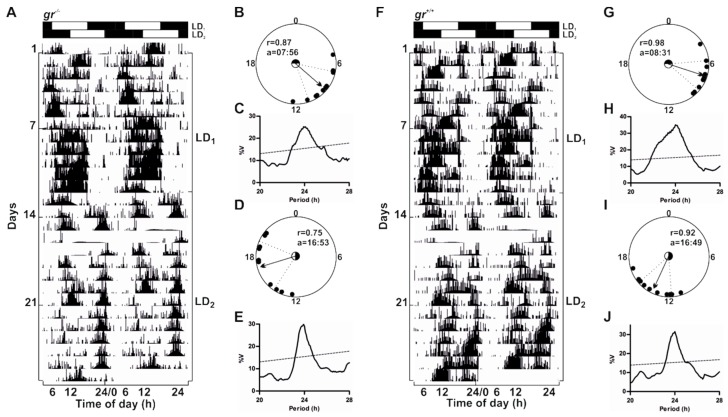
Daily activity rhythms of *gr*^+/+^ and *gr*^−/−^ adult zebrafish. Representative actograms (**A**,**F**), circular diagrams (**B**,**D**,**G**,**I**) and χ^2^ periodogram analysis (**C**,**E**,**H**,**J**) of adult zebrafish *gr*^+/+^ and *gr*^−/−^ subjected to 12:12 LD cycles. In actograms the height of each point represents the number of infrared light-beam interruptions in 10 min. Starting and ending day of each LD cycle (LD_1_ and LD_2_) is shown on the right of the actogram. The number of days is indicated on the left and the time of day is plotted on the bottom of each actogram. For more details, see Figure 2. Circular diagrams showing acrophases for the last 10 days of each LD period are plotted (**B**,**D**,**G**,**I**). Each black dot shows the daily acrophase, while arrows indicate the average acrophase represented as a vector. In each circle the mean vector length (r) and mean acrophase (a) are shown (**B** and **D** for *gr*^−/−^; **G** and **I** for *gr*^+/+^, for LD_1_ and LD_2_, respectively). The circle inside each circular diagram represents critical values of the Rayleigh test (*p* < 0.05) and the black part of the circle shows the duration of dark phase. The dotted lines represent the confidence intervals. Activity records in the last 10 days of each LD cycle were also subjected to χ^2^ periodogram analysis (**C** and **E** for *gr*^−/−^, **H** and **J** for *gr*^+/+^). Confidence limits were chosen at the 99% level.

**Figure 6:**
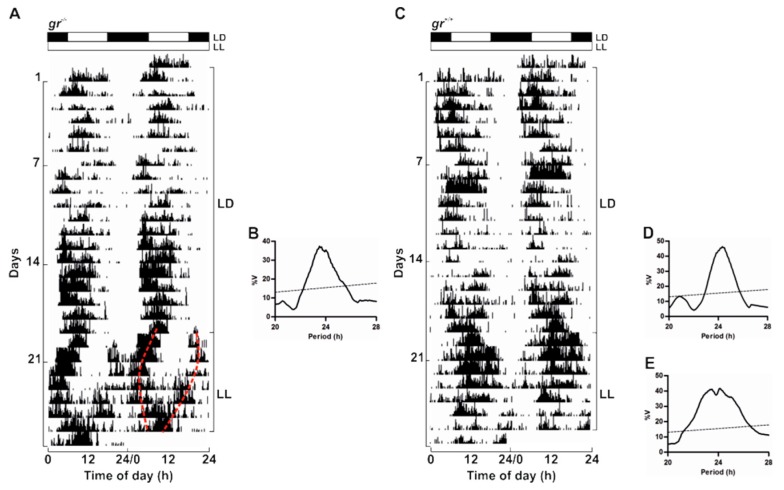
Circadian activity rhythms of *gr*^+/+^ and *gr*^−/−^ adult zebrafish. Representative actograms (**A**,**C**) of adult zebrafish *gr*^+/+^ and *gr*^−/−^ subjected to 12:12 LD cycles and then to LL. Activity records in the last 10 days of LD cycles (**B** and **D**) and of LL (**E**) were subjected to χ2 periodogram analysis. Dotted lines marked the splitting of the rhythm into two independent components with periodicity in the circadian range. For more details, see Figure 2 and Figure 5.

**Figure 7:**
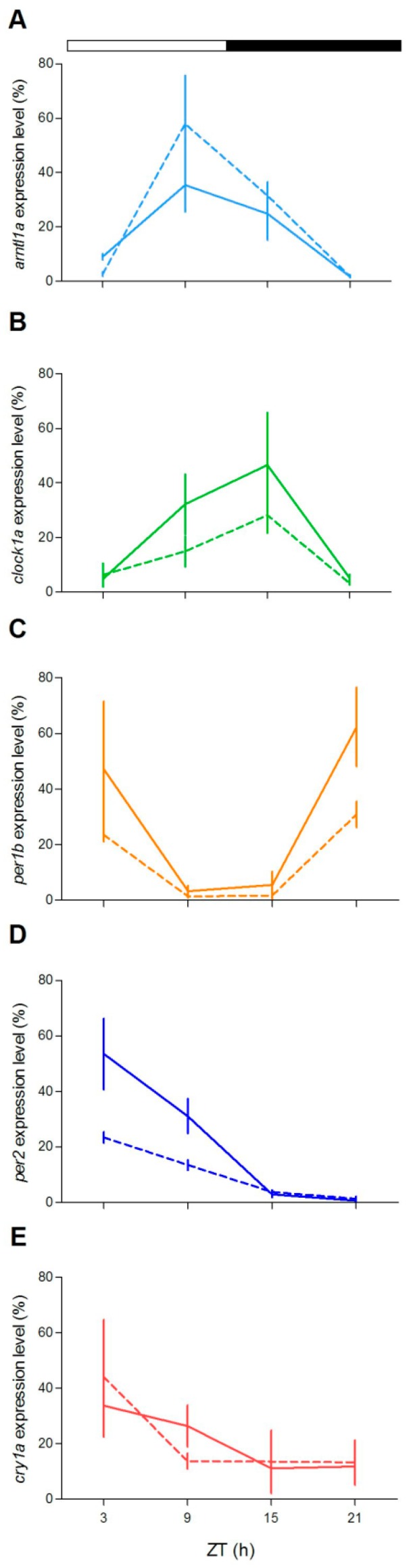
Daily expression levels of clock genes in zebrafish adult eye. qPCR analysis of clock (**A**–**C**) and light-regulated clock (**D**–**E**) gene expression in the eye of zebrafish exposed to LD cycles. For all panels, each point represents the mean ± SEM (n = 4). *gr*^+/+^= solid line; *gr*^−/−^ = dotted line. For more details see Figure 4.

**Figure 8:**
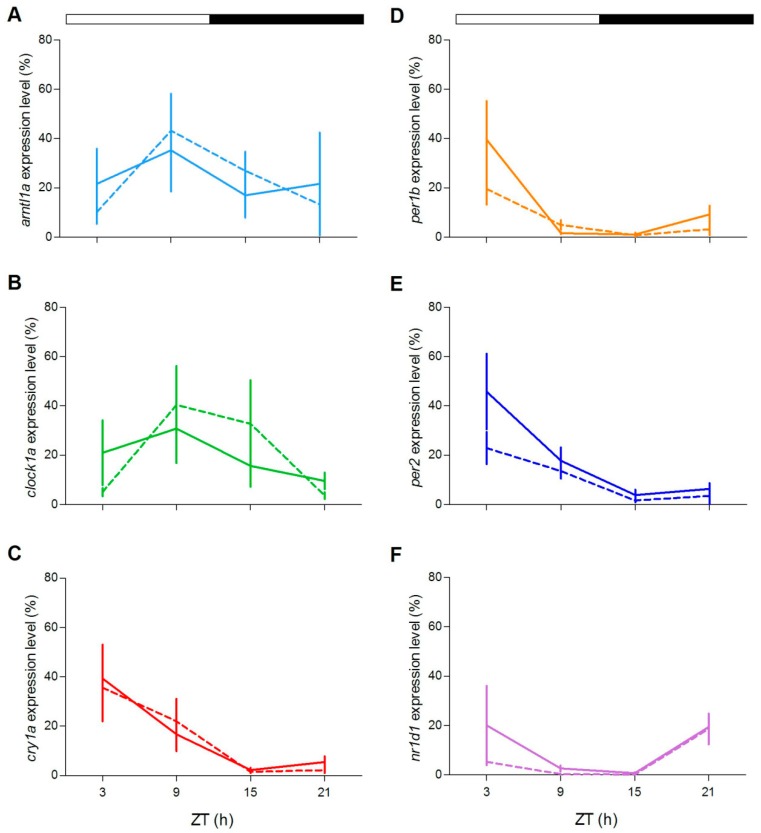
Daily expression levels of clock genes in zebrafish adult liver. qPCR analysis of clock (**A**,**B**,**D**,**F**) and light-regulated clock (**C**,**E**) gene expression in the liver of zebrafish exposed to LD cycles. For all panels, each point represents the mean ± SEM (n = 4). *gr*^+/+^= solid line; *gr*^−/−^ = dotted line. For more details see Figure 4.

**Figure 9:**
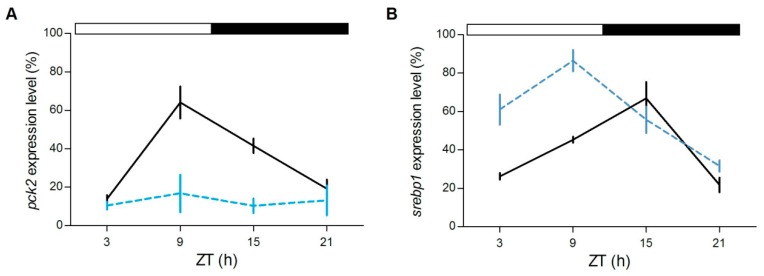
Daily expression levels of genes involved in metabolism in zebrafish adult liver. qPCR analysis of *pck2* (**A**) and *srebp1* (**B**) in the liver of zebrafish exposed to LD cycles. For both panels, each point represents the mean ± SEM (n = 4). *gr*^+/+^= solid line; *gr*^−/−^ = dotted line. For more details see Figure 4.

To verify the presence of behavioural and molecular rhythmicity in zebrafish lacking Gr we performed behavioural and molecular analysis in adults (Figure 5, Figure 6, Figure 7, Figure 8 and Figure 9 and Figure S2). We recorded the locomotor activity in adult zebrafish from both genotypes exposed to two consecutive 12:12 LD cycles (LD_1_: lights-on at 06:00, LD_2_: lights-on at 12:00) (Figure 5). Both mutants and controls showed a clear rhythmic, diurnal pattern (Figure 5A,F and Figure S2), but the mean of daily activity in *gr*^−/−^ was significantly lower than in the control (Mann–Whitney U-test, *p* < 0.001; Figure S2). Periodogram analysis indicated a strong entrainment to both LD cycles (Figure 5C,E,H,J). The 6 h shift of the lights-on from LD_1_ to LD_2_ induced shifts of the activity onsets, which resulted, after 4–5 days, in entrainment to the new light schedule (Figure 5A,E,F,J). To verify the accuracy of the entrained rhythm we estimated the time of acrophase (the time at which the rhythm peak occurred) with respect to the lights on (ZT0) for the last 10 days of recording in each LD cycle. Using a circular statistic approach, we showed that the distribution of acrophases deviated from uniform (Rayleigh test, *p* < 0.002), and the mean acrophases fell between ZT2 and 4 (Figure 5B,D,G,I). As expected, the mean acrophases significantly changed after the 6 h shift of the lights on (*gr*^−/−^: from ZT2 to ZT11; *gr*^+/+^: from ZT2.5 to ZT10.8; Hotelling’s paired test; *p* < 0.0001). 

To confirm the presence of a circadian timekeeping system also in adults, we recorded locomotor activity in *gr* mutant and WT kept in LL after 20 days of entrainment in a 12:12 LD cycle (Figure 6). In LL both genotypes displayed a rhythm of locomotor activity demonstrating the circadian nature of the rhythmicity (Figure 6). As previously reported [37], the locomotor activity in constant lighting conditions exhibited different patterns as a splitting of activity in different periodicity within the circadian range (20–30 h; representative example in Figure 6A) or a unimodal activity with a period close to 24 h (representative example in Figure 6C). Periodogram analysis showed the entrainment to both LD cycles and the significant rhythmicity in LL (Figure 6B,D,E).

In order to explore at the molecular level the circadian clock of both zebrafish genotypes, we examined the expression pattern under LD cycles of a set of clock and clock-related genes in two peripheral oscillators, the eyes and the liver, in adults. In agreement with previous reports, exposure of zebrafish WT to a LD cycle results in robust rhythmic expression of clock genes (Figure 7 and Figure 8; Table S2), with the exception of *clock1a* in the liver. Remarkably, rhythmic gene expression was also encountered in *gr*^−/−^ tissues (Figure 7 and Figure 8, Table S2). Interestingly, the comparison of rhythmic clock gene expression between genotypes show differences in expression level at some ZTs in both tissues (Figure 7C,D: *per1b* and *per2*; Figure 8D,E: *clock1a* and *per2*; pairwise comparison after Kruskal–Wallis test, *p* < 0.05).

Furthermore, in the liver we tested the daily expression of *srebp1* and *pck2*, two genes involved in the lipidic and glucidic metabolism, respectively. *srebp1*, of which the timing of cyclic accumulation in the nucleus is under the control of *nr1d1*, is a key transcription factor that regulates genes in the de novo lipogenesis and glycolysis pathways [38,39], *pck2* codifies for an enzyme essential in gluconeogenesis, a process that is directly regulated by glucocorticoids [40]. Interestingly, while *pck2* is rhythmically expressed only in the *gr*^+/+^ (*gr*^+/+^: *p* < 0.0001, *gr*^−/−^: *p* > 0.7; Figure 9A), *srebp1* changed its expression during the day in both genotypes (*p* < 0.0001; Figure 9B), but peaked at different CTs (*gr*^+/+^: ZT15, *gr*^−/−^: ZT9).

**Figure 10:**
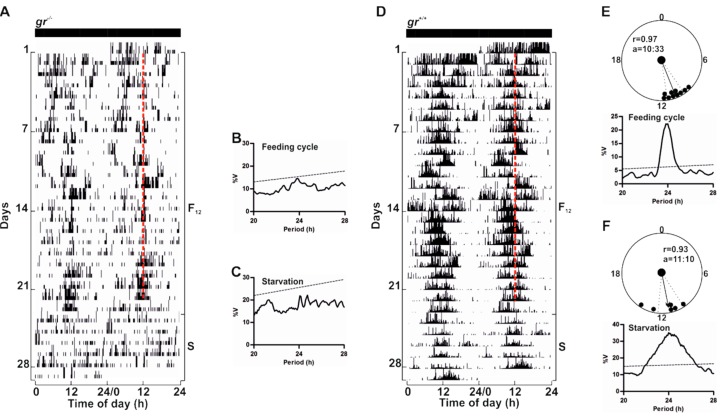
Behavioural entrainment by periodic food availability of *gr*^+/+^ and *gr*^−/−^ adult zebrafish. Representative actograms (**A**,**D**), χ^2^ periodogram analysis and circular diagrams (**B**–**C**,**E**–**F**) of adult zebrafish *gr*^+/+^ and *gr*^−/−^ maintained under constant darkness and fed once a day at a fixed time (12:00). Starting and ending day of each feeding cycle (F_12_) and starvation (S) is shown on the right of the actogram. The number of days is indicated on the left and the time of day is plotted on the bottom of each actogram. Red dotted lines indicate the time of feeding. Circular diagrams showing acrophases for F_12_ and S period are plotted (**E**–**F**). Activity records of each F_12_ or S cycle were subjected to χ^2^ periodogram analysis (**B**–**C**,**E**–**F**). For more details see Figure 5.

To confirm that GC/Gr is not critical for the regulation of circadian rhythmicity in zebrafish, we assessed whether the *gr*^−/−^ zebrafish circadian clock could be entrained by periodic food availability, a second crucial environmental zeitgeber [7]. *gr*^−/−^ and control adults were fed once at the same time each day under constant lighting conditions (Figure 10 and Figure S3). Surprisingly, we did not observe an entrainment of the locomotor activity in *gr*^−/−^ (Figure 10A and Figure S3A). Indeed, mutant adult zebrafish sometimes responded to food administration with an increase of activity at mealtime, but food anticipatory activity (FAA) [20], a characteristic increase in locomotor activity encountered a few hours prior to mealtime, was almost always absent. Periodogram analysis confirmed the arrhythmicity (Figure 10B), also when the time of periodic food availability changed (Figure S3B–D). Furthermore, during starvation, the arrhythmic pattern of locomotor activity showed by adult mutants suggests the absence of regulation by a food-entrainable circadian oscillator (Figure 10A,C). Conversely, in controls we observed the canonical strong entrainment of rhythmic locomotor activity and the FAA (Figure 10D,E). Furthermore, during starvation after feeding entrainment, zebrafish showed a significant circadian rhythmicity (Figure 10D,F).

**Figure 11:**
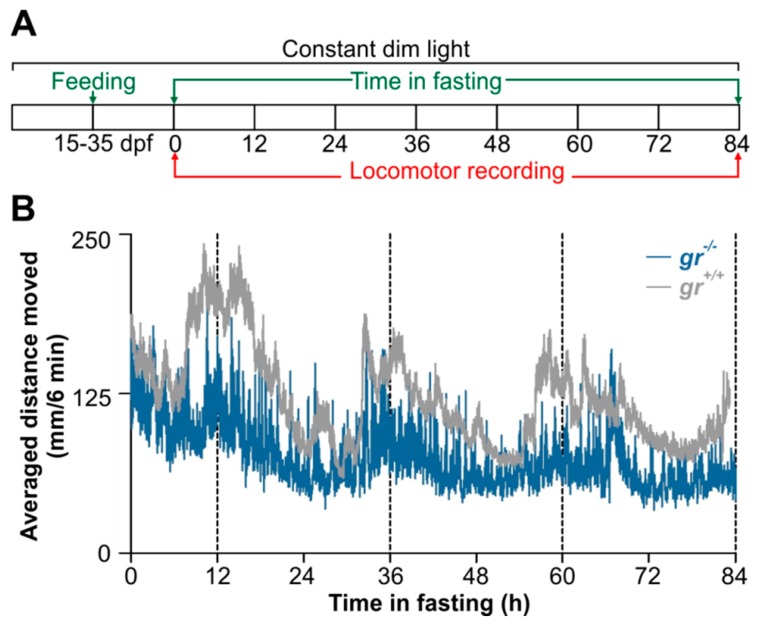
Behavioural entrainment by periodic food availability of *gr*^+/+^ and *gr*^−/−^ zebrafish juvenile. (**A**) Larvae were reared under LL from 15 to 35 dpf and fed once a day at a midday time for 20 days. After then larvae were starved for 84 h and the locomotor activity were recorded. (**B**) Mean waveform of locomotor activity under LL and starvation for 84 h (n = 45/genotype). Vertical dotted lines denote when the feeding would normally have occurred according to the previous regular feeding regime.

This original result motivated us to verify, to our knowledge for the first time, the feeding entrainment also in juveniles. After 20 days of daily feeding at midday (Figure 11A), WT juvenile showed a strong circadian rhythm of locomotor activity (Figure 11B), with a period of 24 h (Cosinor, *p* < 0.001) and a peak at the subjective midday (CT12). By contrast, a weak feeding entrainment has been found in *gr*^−/−^ juvenile (Figure 11B); the circadian activity is dampened in the first two days in constant conditions in comparison with the WT pattern and it became arrhythmic on the third day of recording (Cosinor, *p* > 0.05; Figure 11B). 

**Figure 12:**
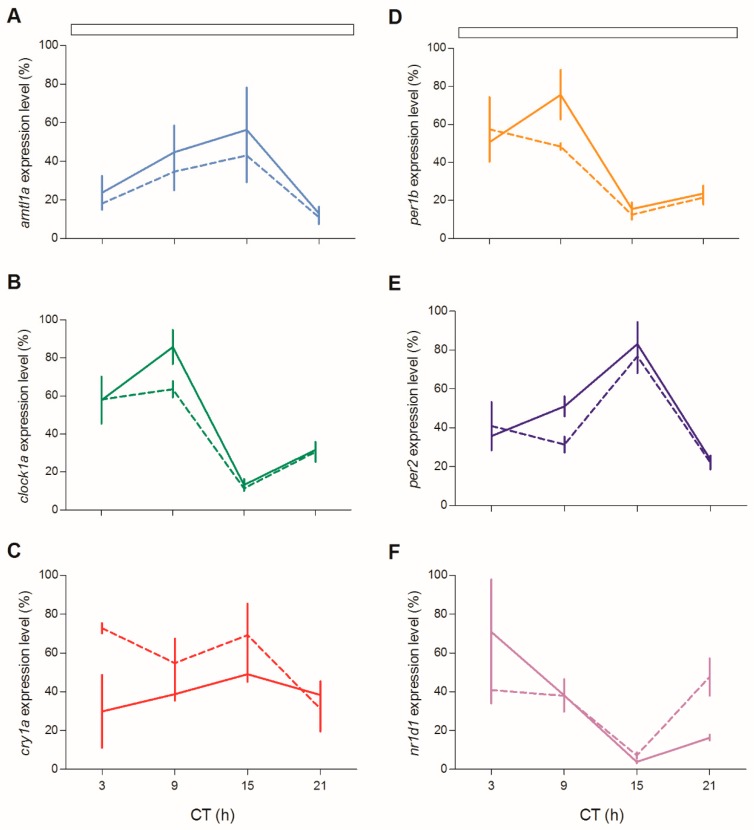
Circadian expression levels of clock genes in zebrafish juvenile. qPCR analysis of clock (**A**,**B**,**D**,**F**) and light-regulated clock (**C**,**E**) gene expression in zebrafish juvenile at the third day of starvation after feeding entrainment. White bars represent LL. For more details see Figure 4.

We next tested circadian clock gene expression in juveniles of both genotypes during the third day of fasting after feeding entrainment (Figure 12; Table S2). Rhythmic clock gene expression was observed for all clock genes (Figure 12A–D,F), with the only exception of *cry1a* (Figure 12E). Furthermore, the level of expression of *per1b* and *clock1a* was significantly dampened during the subjective day (Figure 12B,C; pairwise comparison after Kruskal–Wallis test, *p* < 0.05).

## 4. Discussion

Fish are valuable complementary animal models for studying various aspects of clock biology. Although many clock genes are present in multiple copies, the molecular core clock mechanism is fundamentally conserved and the most important environmental *zeitgebers*, such as light and food, are able to entrain the circadian clock [7,18]. Furthermore, since their peripheral clocks are directly entrained by light in almost all tissues investigated [41,42,43], and cell lines can be synchronized by metabolic shock as a serum treatment [20], fish have become interesting models for studying how clocks respond to environmental cues and endogenous inputs, as neuropeptides and hormones.

Here we explored the role of the Gr/GCs complex in the zebrafish circadian clock system. Using zebrafish Gr null mutants we showed that Gr/GCs did not play a role in the generation of the circadian rhythmicity and in the photic entrainment. Indeed, both larvae and adults’ behavioural activity and clock gene expression were entrained to the LD cycle and rhythmic in constant conditions. Daily activity rhythm was also obtained by Griffiths and coworkers [44] with 5 dpf larvae from a different zebrafish *gr* mutant line, named *gr^s357/s357^* [44], in which DNA binding activity of the Gr has been abolished by a single base-pair substitution in the DNA-binding domain. Interestingly, differences in the pattern of expression of clock genes in *gr*^−/−^ larvae and adult tissues indicate a modulatory role of GCs on both positive and negative elements of the circadian feedback loops [16]. Previous investigation in the liver of goldfish *Carassius auratus* indicated that *per1*, *clock1a* and *bmal1a* are glucocorticoid targets and GREs have been identified in clock gene promoters [24]. We found that expression of the light-inducible gene *per2* in *gr*^−/−^ is dampened. Both *per2* and *cry1a* expression is light-induced via the D-box and E-box elements [7,45,46], but their promoters contain GRE and negative GRE that may enhance or repress their transcription [46].

Previous analysis of liver transcriptome showed a daily and rhythmic expression pattern of many genes related to metabolic processes [47]. Among them we examined *pck2* and *srebp1*, two clock-regulated genes involved in glucidic and lipidic metabolism [48,49]. Pck2 catalyzes the conversion of oxaloacetate to phosphoenolpyruvate and its transcription is enhanced by GC [40]. In *gr*^−/−^ zebrafish liver we found that the daily expression of *pck2* is abolished and its levels are markedly reduced confirming the positive effect of GCs on hepatic gluconeogenesis also in fish. By contrast, the expression of *srebp1* is rhythmic in both zebrafish genotypes, but the acrophase was anticipated in *gr*^−/−^. This change in the peak of expression from the beginning of the dark phase to the end of the light phase could be related to the significant dampening of *nr1d1* expression at ZT3 found in the mutant. *Rev-erbα* (*nr1d1*) is member of the secondary loop and acts as repressor of *bmal1* (*arntl1*) transcription [9]. It is highly expressed in metabolic tissues, with known functions in conferring circadian clock integration to glucose, lipoprotein and bile acid metabolism [50]. A previous investigation in mice showed that *rev-erbα* is a key player in the circadian regulation of cholesterol synthesis by influencing rhythmic Srebp activity [38]. We could propose that the anticipation in the acrophase of *srebp1* depends to the reduction of the *nr1d1* expression levels at the beginning of the light phase.

The link between metabolic signals and circadian system is also demonstrated by the fact that the feeding is temporally organized during the day and it is regulated by the circadian clock [16,18]. Feeding entrainment has been found in many marine and freshwater fish species, including blind cavefish which lack the LEO, but maintain the FEO [20]. Our investigation in *gr*^−/−^ confirm the importance of GCs in the feeding entrainment also in fish. As previously shown, adult WT activity was entrained to daily food availability and showed an evident FAA. Conversely, adult *gr*^−/−^ were not synchronized by daily food administration. To confirm this novel result, we adapted the adult zebrafish feeding protocol to larvae/juveniles. Also at this developmental stage, a strong entrainment was present only in WT. To our knowledge this is the first evidence of the existence of feeding entrainment of locomotor activity in early juvenile zebrafish. It confirms the importance of feeding signal also during the juvenile stages. In addition, the absence of feeding entrainment in adult *gr*^−/−^ clearly points to the role of GC in the regulation of the feeding entrainment of the circadian clock in fish. However, we showed that, after one month of regular daily food administration, clock genes in *gr*^−/−^ early juveniles are rhythmically expressed, with the exception of *cry1a*, indicating that the role of GCs in the zebrafish feeding entrainment could be not linked directly to the clock gene expression. Differently, in mammals the GC rhythmicity is an internal periodic signal to the peripheral tissues that, through Gr, modulates the transcription of clock genes as *per1*, *per2* and *rev-erbα* [12]. However, as the role of GC/Gr could be limited to particular brain nuclei, further detailed investigations are necessary to clarify the neural control on the feeding entrainment in fish.

The circadian system of vertebrates is a multioscillatory network comprising a master clock often located in the hypothalamus and many peripheral clocks in different organs and tissues. Light and food availability oscillate in a periodic and predictable manner in the natural environment and are the most widespread *zeitgeber* that entrain circadian clocks. Taken together, our results confirmed in a non-mammalian species, the zebrafish, the role of GCs as endocrine signals related to the feeding synchronization of circadian clocks.

We also detected, in *gr*^−/−^ larvae, a delay of the daily locomotor rhythmicity onset resulting in rhythmic activity one day later with respect to control. This delay could be related, at least partially, to a general slowdown in mutant muscle development as suggested by birefringence analysis of *gr*^−/−^ larvae muscle during development. This result is also in agreement with previous *gr* knockdown studies [51,52] showing an involvement of GCs on mesoderm development. Moreover, delayed physical development leading to reduced locomotor activity was also found at 5 dpf by Wilson and coworkers after *gr* morpholino treatment [53]. A reduction in spontaneous activities was detected in *gr^s357^* mutant larvae analyzed for a 24 h recording period [44]. The decrease in locomotor activity was associated to behavioural changes linked to depression or anxiety-like states [44]. Locomotor activity was partially restored by treatment with the antidepressant fluoxetine, possibly through modulation of HPA axis hyperactivity of the mutant line [44,54].

Although the mechanism remains to be discerned, our results confirmed the role of GCs-mediated Gr signaling in the feeding entrainment in a non-mammalian species, the zebrafish. Thus zebrafish *gr* mutant is an excellent model to study the role of GCs and Gr in the circadian timekeeping system and to deepen our knowledge on the pathways involved in feeding behaviour.

## Supplementary Materials

The following are available online at https://www.mdpi.com/2073-4409/8/11/1342/s1.
