# Supplementary material for: Feeding Entrainment of the Zebrafish Circadian Clock Is Regulated by the Glucocorticoid Receptor
Source: Cells. 2019 Oct 29;8(11):1342. doi: 10.3390/cells8111342 (PMC6912276; doi:10.3390/cells8111342)
Supplement: Supplementary file 1 [file cells-08-01342-s001.zip › Morbiato et al_Supplementary tables and figures/Figure S3 11-10-19.pdf]

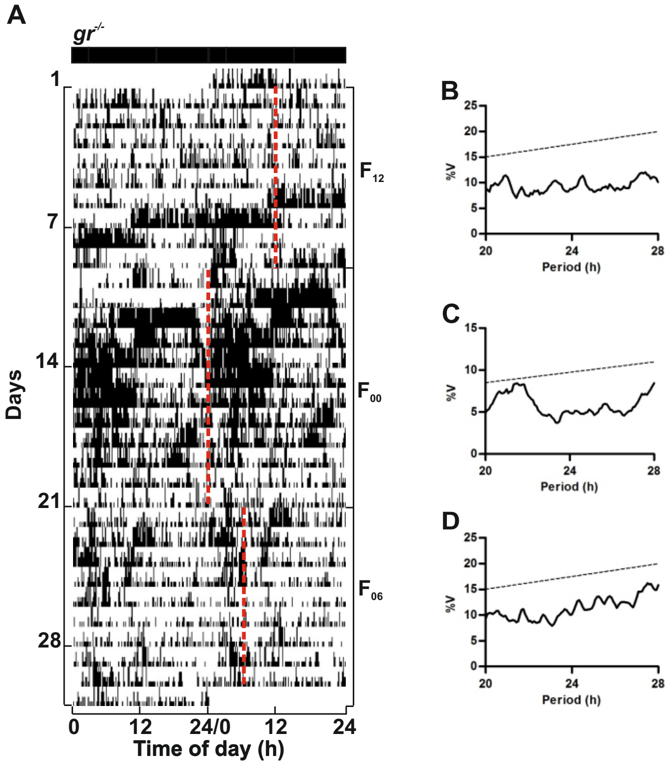

Figure S3.

Behavioral entrainment by periodic food availability of *gr*$^{+/+}$ and *gr*$^{-/-}$ adult zebrafish. Representative actograms (**A**) and χ^2 periodogram analysis (**B-D**) of adult zebrafish *gr*$^{+/+}$ and gr$^{-/-}$ maintained under constant darkness and fed once a day at a fixed time (06:00, 12:00, 24:00). Starting and ending day of each feeding cycle (F_{00}, F_{12} and F_{06}) and starvation (S) is shown on the right of the actogram. The number of days is indicated on the left and the time of day is plotted on the bottom of each actogram. Activity records of each F cycle were subjected to χ^2 periodogram analysis (**B-D**). Red dotted line indicate the time of feeding. For more details see Fig. 5 and 10.
