# Supplementary material for: Feeding Entrainment of the Zebrafish Circadian Clock Is Regulated by the Glucocorticoid Receptor
Source: Cells. 2019 Oct 29;8(11):1342. doi: 10.3390/cells8111342 (PMC6912276; doi:10.3390/cells8111342)
Supplement: Supplementary file 1 [file cells-08-01342-s001.zip › Morbiato et al_Supplementary tables and figures/Table S1 06 09 19.pdf]

Table S1
qPCR primers

Gene	Forward (5'-3')	Reverse (5'-3')	Genbank acc. no.
clock1a	CTGGAGGATCAGCTGGGTAG	CACACACAGGCACAGACACA	NM_130957
arntl1a	TAGAGCGCTGTTTGCTGATG	GACCCGTGGACTTCAGTGAC	NM_131578
per1b	CCGTCAGTTTCGCTTTTCTC	ATGTGCAGGCTGTAGATCCC	NM_001030183
per2	ATGTCGATGGCTTTAGGCAG	CGAGACATCCAGAAGGTGCT	NM_182857
cry1a	TCCGCTGTGTGTACATCCTC	CAAACACTGCAGCAAAAACC	NM_001077297
nr1d1	GCAATTCACCCAACAAATCAG	CAGGCATGGACGCCATAGT	NM_001130592
pck2	CTGTGTGCTCATCCAAACTCC	GATCTCATAGCTGCACCAACG	NM_213192
srebp1	GACACTTCTCTGGACACTCTG	ATCGAACAGCCCAAACTCC	NM_001105129
rpl13a	TCTGGAGGACTGTAAGAGGTATGC	AGACGCACAATCTTGAGAGCAG	NM_198143
gapdh	GTGGAGTCTACTGGTGTCTTC	GTGCAGGAGGCATTGCTTACA	NM_001115114.1
rplp0	CTGAACATCTCGCCCTTCTC	TAGCCGATCTGCAGACACAC	NM_001161350
18s	ACCACCCACAGAATCGAGAAA	GCCTGCGGCTTAATTTGACT	KY486501
