# Supplementary material for: Feeding Entrainment of the Zebrafish Circadian Clock Is Regulated by the Glucocorticoid Receptor
Source: Cells. 2019 Oct 29;8(11):1342. doi: 10.3390/cells8111342 (PMC6912276; doi:10.3390/cells8111342)
Supplement: Supplementary file 1 [file cells-08-01342-s001.zip › Morbiato et al_Supplementary tables and figures/Figure S1_26 09 19.pdf]

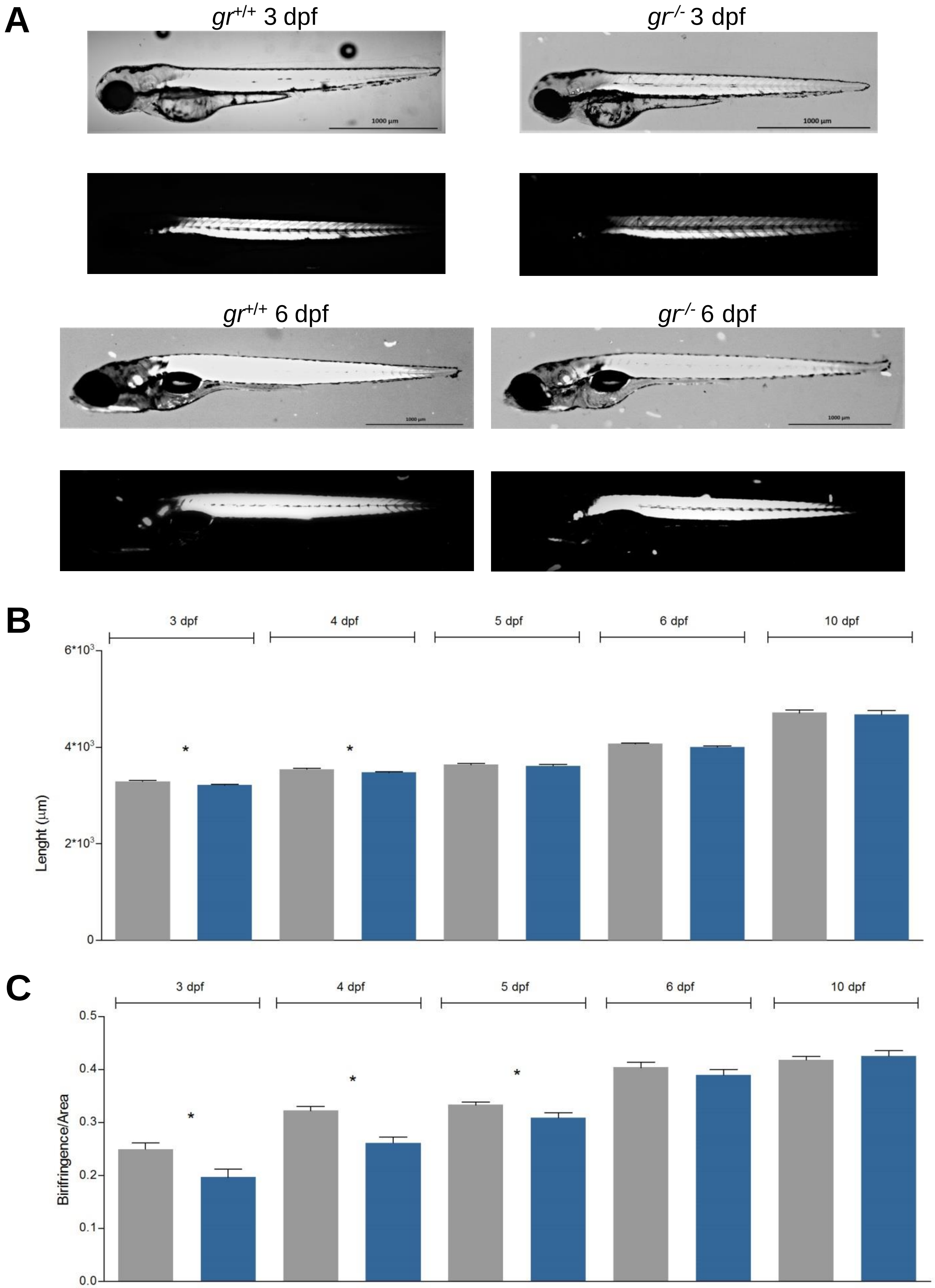

Figure S1

(**A**) Representative images under normal and polarized light of 3 and 6 dpf live *gr*$^{+/+}$ and *gr*$^{-/-}$ larvae. Under polarized light, the muscle of *gr*$^{+/+}$ 3 dpf siblings appears brighter than that of *gr*$^{-/-}$ at the same age due to a reduction in birefringence. (**B**) Body length analysis of both genotypes between 3 to 10 dpf. Data presented as mean ± SEM. (**C**) Quantification of embryo trunk muscles birefringence showing a statistically significant reduction in *gr*$^{-/-}$ at 3, 4 and 5 dpf. The birefringence is similar at 6 and 10 dpf. * = $p<0.05$.
