# Supplementary material for: Feeding Entrainment of the Zebrafish Circadian Clock Is Regulated by the Glucocorticoid Receptor
Source: Cells. 2019 Oct 29;8(11):1342. doi: 10.3390/cells8111342 (PMC6912276; doi:10.3390/cells8111342)
Supplement: Supplementary file 1 [file cells-08-01342-s001.zip › Morbiato et al_Supplementary tables and figures/Table S2 26 09 19.pdf]

Table S2
Statistical analysis

Larvae – Light entrainment

	5 dpf			
	gr$^{+/+}$		*gr*$^{-/-}$	
	P	A	P	A
arntl1	<0.00001	9	<0.00001	15
clock1a	<0.00001	15	<0.00001	15
per1b	0.007	3	<0.00001	3
per2	0.003	3	-	3
cry1a	<0.00001	3	<0.00001	3
	6 dpf			
	gr$^{+/+}$		*gr*$^{-/-}$	
	P	A	P	A
arntl1	<0.00001	15	<0.00001	15
clock1a	<0.00001	15	<0.00001	15
per1b	<0.00001	21	0.001	3
per2	-	3	<0.00001	3
cry1a	0.001	3	<0.00001	3
	12 dpf			
	gr$^{+/+}$		*gr*$^{-/-}$	
	P	A	P	A
arntl1	0.001	9	<0.00001	15
clock1a	0.005	9	<0.00001	15
per1b	0.006	3	<0.00001	21
per2	<0.00001	3	0.002	3
cry1a	<0.00001	3	0.013	3

Adults – Light entrainment

	Eyes			
	gr$^{+/+}$		*gr*$^{-/-}$	
	P	A	P	A
arntl1	<0.00001	9	<0.00001	9
clock1a	0.003	15	0.008	15
per1b	<0.00001	21	<0.00001	21
per2	<0.00001	3	<0.00001	3
cry1a	<0.00001	3	<0.00001	3
	Liver			
	gr$^{+/+}$		*gr*$^{-/-}$	
	P	A	P	A
arntl1	-	6	-	6
clock1a	-	9	-	9
per1b	<0.00001	3	<0.00001	3
per2	<0.00001	3	<0.00001	3
cry1a	<0.00001	3	<0.00001	3
nr1d1	<0.00001	21	0.001	21

Juvenile – Feeding entrainment

	35 dpf			
	gr$^{+/+}$		*gr*$^{-/-}$	
	P	A	P	A
arntl1	0.023	15	0.006	15
clock1a	<0.00001	9	<0.00001	9
per1b	<0.00001	9	0.002	3
per2	<0.00001	15	0.015	15
cry1a	-	15	-	3
nr1d1	-	21	0.006	3
