# Supplementary material for: Feeding Entrainment of the Zebrafish Circadian Clock Is Regulated by the Glucocorticoid Receptor
Source: Cells. 2019 Oct 29;8(11):1342. doi: 10.3390/cells8111342 (PMC6912276; doi:10.3390/cells8111342)
Supplement: Supplementary file 1 [file cells-08-01342-s001.zip › Morbiato et al_Supplementary tables and figures/Figure S2_06 09 19.pdf]

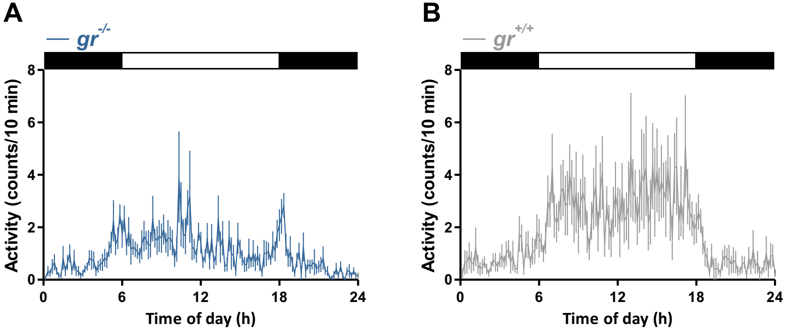

Figure S2.

Mean waveforms of gr-/- (A) and gr+/+ (B) zebrafish entrained to LD cycle are represented. Each point in the mean waveform has been calculated as the mean ± SEM from 10 min binned data across all LD1 cycle (12 days) shown on actograms from Fig. 5.
